# Increased Risk for Hip Fractures among Patients with Cholangitis: A Nationwide Population-Based Study

**DOI:** 10.1155/2018/8928174

**Published:** 2018-06-05

**Authors:** Chieh-Cheng Hsu, Horng-Chaung Hsu, Che-Chen Lin, Yu-Chiao Wang, Hsuan-Ju Chen, Yung-Cheng Chiu, Chien-Chun Chang, Shu-Jui Kuo

**Affiliations:** ^1^Department of Orthopaedic Surgery, Kaohsiung Chang Gung Memorial Hospital and Chang Gung University College of Medicine, Kaohsiung 83301, Taiwan; ^2^Department of Orthopedic Surgery, China Medical University Hospital, Taichung, Taiwan; ^3^College of Medicine, China Medical University, Taichung, Taiwan; ^4^Management Office for Health Data, China Medical University Hospital, Taichung, Taiwan

## Abstract

**Background:**

Cholangitis is the infectious disease involving the biliary tract, which may induce systemic inflammation. Bone loss is a well-known sequelae after systemic inflammatory disease, and one grave complication after osteoporosis is hip fracture. We want to know whether cholangitis can contribute to increased risk of hip fracture.

**Methods:**

All the patients diagnosed with cholangitis since January 1, 2001, to December 31, 2009, were assessed. All the subjects with cancer history, traumatic accident, and previous fracture were excluded. We selected the controls without cholangitis and matched the controls to cholangitis patients by age, sex, osteoporosis, and the use of steroid for more than 30 days by approximately 1:4 ratio.

**Results:**

There were 2735 subjects in the cholangitis cohort and 10915 in the noncholangitis cohort. There were 101 hip fractures in the cholangitis cohort with the incidence density of 7.58 per 1000 person-years. As for the noncholangitis cohort, 366 individuals suffered from hip fracture with the incidence density of 5.86 per 1000 person-years. The risk of hip fracture was higher in the cholangitis cohort with a 1.29-fold increased risk than the noncholangitis cohort (hazard ratio = 1.29, 95% confidence interval = 1.03-1.61). The association between cholangitis and the hip fracture was more prominent among subjects less than 65 years (hazard ratio = 2.65, 95% confidence interval =1.30-5.39) and the subjects without comorbidities (hazard ratio = 3.01, 95% confidence interval = 1.42-6.41).

**Conclusions:**

Cholangitis is associated with higher risk for hip fracture, especially among young subjects free from medical comorbidities.

## 1. Introduction

Cholangitis is the infectious disease affecting the biliary tract [1]. It is mainly caused by the obstruction of biliary tract, mostly by the stone [2]. Cholangitis has been considered as a risk factor for subsequent gastrointestinal cancer because of the systemic inflammation triggered by the disease [3]. However, the detrimental consequences of systemic inflammation after cholangitis other than cancer have not been completely delineated at present.

Bone loss is a well-known sequelae after systemic inflammatory disease, which can be explained by the perspective of evolutionary medicine, altered energy expenditure and storage, and neuroendocrine as well as immunologic factors [4]. One grave complication after osteoporosis is hip fracture [5]. Hip fractures account for one-fourth of geriatric fractures necessitating hospitalization with substantial morbidity and mortality [6].

Bone complications are prevalent among patients with liver dysfunction. The known liver diseases associated with bone loss included chronic hepatitis C infection, primary biliary cirrhosis, primary sclerosing cholangitis, or liver transplant. The incidences of osteoporosis and fracture risk are high among these liver diseases [7]. However, the bone disease after cholangitis has not been discussed before. Based on the observations mentioned above, we hypothesize that cholangitis could lead to increased risk for hip fractures. We thus performed a nationwide population-based study to ascertain our hypothesis.

## 2. Materials and Methods

The Taiwanese government established the nationwide and single-payer based National Health Insurance (NHI) program for all Taiwanese citizens. The National Health Research Institute (NHRI) constructed the National Health Insurance Research Database (NHIRD) comprising the claim data, registry of beneficiary, and clinic and hospital files of the insured individuals. In our study, we set up the study cohort by the Longitudinal Health Insurance Database (LHID), which was a subset of NHIRD. The ICD-9-CM (International Classification of Diseases, 9th Revision, Clinical Modification) system was utilized as the disease coding system in NHIRD. The NHRI removed the original identification number to safeguard the privacy of all the insured individuals and provided a scrambled identification number to link the claim data to each insured citizen before releasing the data for researchers.

The study was approved by the Ethics Review Board of China Medical University (CMUH104-REC2-115).

We constructed a population-based cohort study to determine whether the patients with cholangitis are more susceptible to hip fractures. All of the patients who had the admission episode because of the de novo diagnosis of cholangitis (ICD-9-CM 576.1) since January 1, 2001, to December 31, 2009, were all assessed. The subjects with cancer history (ICD-9-CM 140-208), accident indicative of high energy trauma (presence of E coding), and previous hip fracture history (ICD-9-CM 820) were all excluded.

The index date was defined as the date when the diagnosis of cholangitis was initially coded.

We selected the control subjects without the coding of cholangitis from the LHID and matched the controls to cholangitis patients by age (per 5 year), sex, the coding of osteoporosis (ICD-9-CM 733.0 and 733.1) at index date, the continuous use of oral steroid over 30 days before the index date, and year of index date by approximately 1:4 ratio. The diagnosis of osteoporosis would only be coded by the Taiwanese physician if the patient had the T score value of less than -2.5. The control subjects fulfilling the matching criteria were enrolled on the same date as the matched cholangitis patients.

The two sample* t*-test for continuous variables and chi-square test for categorical variables were utilized for between-group comparisons. The incidence density of the hip fracture was expressed as case per 1000 person-years. The Kaplan-Meier method was utilized to measure the cumulative incidence of hip fractures, and the log rank test was utilized to determine the significance of between-group differences. To interpret the risk of hip fracture for both cohorts, the crude and adjusted hazard ratios (HRs) and 95% confidence intervals (CIs) were evaluated by utilizing the single-variable and multivariable Cox proportional hazard models. The SAS 9.4 software (SAS Institute, Cary, NC, USA) was utilized for data analysis and the R software (R Foundation for Statistical computing, Vienna, Austria) was applied to plot the incidence curves.

## 3. Results

**Figure 1:**
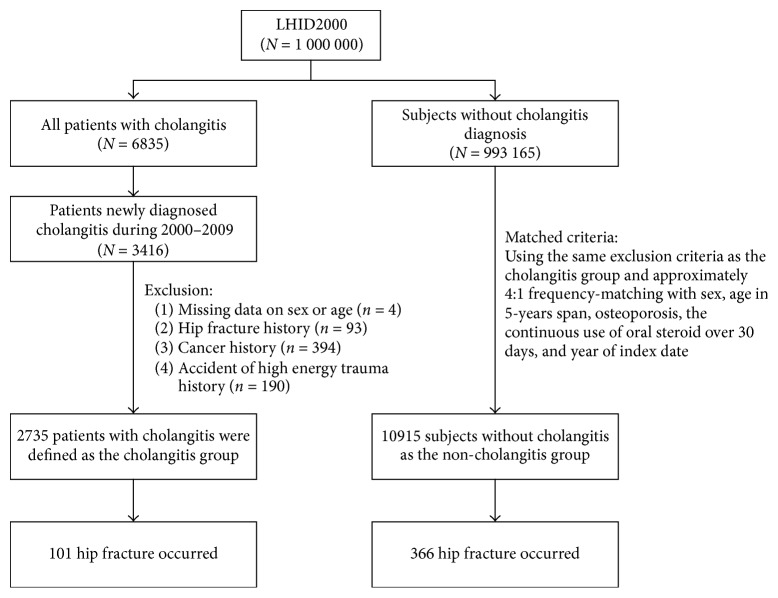
The flow diagram of the recruitment process.

**Figure 2:**
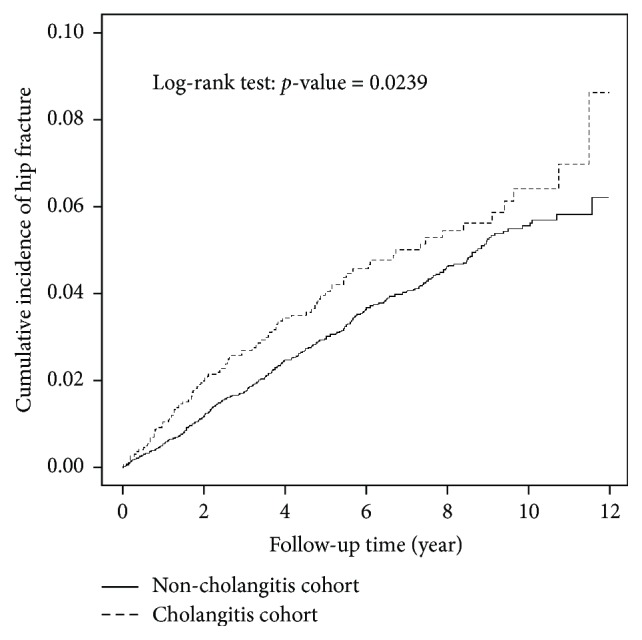
The incidence of hip fracture among cholangitis and control cohorts. The dashed line indicates the cholangitis cohort, and the solid line indicates the matched control cohort. The log rank test was utilized to assess the significance of difference between the curves.

There were 2735 patients in the cholangitis cohort and 10915 subjects in the noncholangitis cohort (Table 1). The distribution of age, sex, history of osteoporosis, and oral steroid use were similar between the two groups. The enlisted comorbidities, including hypertension, diabetes, epilepsy, ischemic heart disease, chronic obstructive pulmonary disease, anxiety, sleep disorder, stroke, liver cirrhosis, and end stage renal disease were all higher in the cholangitis cohort than in the control cohort (all p < 0.05). The flow diagram for subject recruitment was shown in Figure 1. There were 101 hip fractures in the cholangitis cohort with the incidence density of 7.58 per 1000 person-years. As for the noncholangitis cohort, 366 individuals suffered from hip fracture with the incidence density of 5.86 per 1000 person-years (Table 2). The cholangitis cohort had higher hip fracture incidence than in the noncholangitis cohort (Figure 2, p = 0.02). After adjustment for age, sex, and comorbidities, the cholangitis patients had 1.29-fold increased risk of hip fracture than the noncholangitis subjects (hazard ratio = 1.29, 95% confidence interval = 1.03-1.61). Age, female gender, diabetes, stroke, and end stage renal disease were also associated with higher incidence of hip fracture in our model.

Table 3 showed that the association between cholangitis and the increased risk of hip fracture was more prominent among subjects aged less than 65 years (hazard ratio = 2.65, 95% confidence interval =1.30-5.39) and the subjects without comorbidities (hazard ratio = 3.01, 95% confidence interval = 1.42-6.41).

## 4. Discussion

In our study, we demonstrated that the admission episode for more than seven days due to de novo cholangitis is associated with higher risk for hip fracture. We also showed that the association between cholangitis and the increased risk of hip fracture was more prominent among subjects aged less than 65 years and the subjects without comorbidities. These results have not been reported before.

Rainer et al. have proposed the “three-pillar theory” to explain the bone loss due to chronic systemic inflammation, including evolutionary medicine, altered energy expenditure and storage, and neuroendocrine as well as immunologic factors [4]. Among the immunologic factors related to chronic systemic inflammation, the receptor activator of nuclear factor-*κ*B (RANK) is well known for its role on the osteoclastic activation and the pathogenesis of osteoporosis [4]. One of the most devastating complications of osteoporosis is hip fracture, which is associated with significant morbidity and mortality [8]. It is thus prudent to speculate the correlation between the disease entity triggering systemic inflammation, such as cholangitis, and the occurrence of hip fracture.

The fever component of Charcot triad (jaundice, right upper quadrant abdominal pain, and fever) highlights the fact that cholangitis can initiate systemic inflammatory response. It is intriguing that whether the magnitude and duration of systemic inflammation of cholangitis can contribute to bone loss, or even hip fracture.

Angulo et al. performed 10-year longitudinal bone mineral density assay for patients with primary sclerosing cholangitis (PSC) for 237 patients. They demonstrated that osteoporosis was found in 15% patients and occurred 23.8-fold more frequently than the matched controls. Patients with PSC lost 1% of bone mass per year [9]. These results suggested that the inflammation of biliary tract correlates with bone loss, and the inflammation of biliary tract can exert the detrimental effect on bone mass in a time-dependent manner. In our study, we showed that the patients with cholangitis had 1.29 times the risk for hip fracture when compared with the matched control subjects.

Age (more than 65-year-old), female gender, stroke, and ESRD were also associated with higher incidence of hip fracture in our model. The findings derived from our model were consistent with previous studies [10, 11]. These consistent results not only supplement the published observations but also consolidate the validity and internal consistency of our model.

There are limitations to the study. First, not all the risk factors for the hip fracture can be obtained from the NHIRD for analysis, such as vitamin D insufficiency. The diagnosis of osteoporosis would only be coded by the Taiwanese physician if the patient had the T score less than -2.5. The true value of the T score cannot be obtained from the database. We cannot exclude the possibility that some subjects can have low-grade systemic inflammation triggered by subclinical stone disease, which was designated into the control group. However, the presence of subclinical cholangitis will contribute to the underestimation of the significance of difference. The best way to evaluate the pure impact of cholangitis on the risk of hip fracture was propensity score matching for all medical comorbidities. The lack of propensity score matching may raise the concern that the observed higher facture rate in the cholangitis cohort may be secondary to some other concomitant medical comorbidities associated with cholangitis. However, propensity score matching will jeopardize the generalizability of our results, so we just match the coding of osteoporosis and the history of steroid use only.

It is interesting that the effect of cholangitis on the occurrence of hip fracture was more prominent among subjects aged less than 65 years and the subjects without baseline comorbidities. We assume that the systemic inflammatory burden was higher among the elderly subjects and the subjects with medical comorbidities. The process of cholangitis would only exert mild increments on the systemic inflammatory burden. However, among the subjects with lower baseline systemic inflammation, the inflammatory effects following cholangitis will be more prominent.

## 5. Conclusions

In conclusion, we showed that cholangitis is associated with higher risk for hip fracture, especially among the subjects without baseline medical comorbidities and aged less than 65 years. Preventive measures for hip fracture, such as antiosteoporotic regimen, may be warranted for the young cholangitis patients without medical comorbidities.

## Figures and Tables

**Table 1:** Comparison of demographic profiles and history of comorbidities in cholangitis and noncholangitis cohorts.

	Cholangitis				*p *value
	No (N=10915)		Yes (N=2735)		
	n	%	n	%	
Sex					0.99
Women	5163	47.3	1294	47.3	
Men	5752	52.7	1441	52.7	
Age, year					0.99
<35	580	5.31	145	5.30	
35–65	4560	41.8	1140	41.7	
≥65	5775	52.9	1450	53.0	
Mean (SD) ^#^	63.7 (16.5)		63.9 (16.5)		0.58
Comorbidities					
Hypertension	5411	49.6	1524	55.7	<.0001
Diabetes	2285	20.9	811	29.7	<.0001
Epilepsy	110	1.01	48	1.76	0.0011
IHD	2983	27.3	936	34.2	<.0001
COPD	2174	19.9	663	24.2	<.0001
Stroke	2127	19.5	689	25.2	<.0001
Live cirrhosis	172	1.58	235	8.59	<.0001
Osteoporosis	1650	15.1	417	15.2	0.8653
ESRD	82	0.75	31	1.13	0.0485
Drug used					
Steroid	3308	30.3	831	30.4	0.9376

Chi-square test; ^#^Student's *t*-test.

IHD: ischemic heart disease; COPD: chronic obstructive pulmonary disease.

ESRD: end-stage renal disease; SD: standard deviation.

**Table 2:** The adjusted hazard ratio of hip fracture in different risk factors.

Variables	N	Event	Crude HR (95%CI)	Adjusted HR^†^ (95% CI)
Cholangitis				
No	10915	366	1.00	1.00
Yes	2735	101	1.28 (1.03-1.61)	1.29 (1.03-1.61)
Sex				
Women	6457	279	1.00	1.00
Men	7193	188	0.63 (0.53-0.76)	0.64 (0.53-0.79)
Age	13650	467	1.12 (1.11-1.13)	1.11 (1.10-1.12)
Comorbidities				
Hypertension				
No	6715	120	1.00	1.00
Yes	6935	347	3.26 (2.65-4.01)	1.07 (0.84-1.34)
Diabetes				
No	10554	316	1.00	1.00
Yes	3096	151	1.87 (1.54-2.27)	1.23 (1.00-1.5)
Epilepsy				
No	13492	462	1.00	1.00
Yes	158	5	1.25 (0.52-3.01)	0.83 (0.34-2.01)
IHD				
No	9731	251	1.00	1.00
Yes	3919	216	2.47 (2.06-2.97)	0.98 (0.80-1.21)
COPD				
No	10813	307	1.00	1.00
Yes	2837	160	2.39 (1.98-2.90)	1.13 (0.92-1.40)
Stroke				
No	10834	287	1.00	1.00
Yes	2816	180	2.96 (2.45-3.56)	1.26 (1.03-1.55)
Live cirrhosis				
No	13243	452	1.00	1.00
Yes	407	15	1.53 (0.92-2.56)	1.43 (0.85-2.40)
Osteoporosis				
No	11583	331		
Yes	2067	136	2.56 (2.09-3.12)	1.06 (0.85-1.33)
ESRD				
No	13537	460	1.00	1.00
Yes	113	7	2.89 (1.37-6.11)	3.07 (1.45-6.52)
Steroid				
No	9511	303	1.00	1.00
Yes	4139	164	1.51 (1.25-1.83)	1.10 (0.9-1.35)

IHD: ischemic heart disease; COPD: chronic obstructive pulmonary disease.

ESRD: end-stage renal disease.

HR, hazard ratio; adjusted HR^†^: multiple analysis including age, sex, each comorbidity, and oral steroid used.

**Table 3:** Incidence and adjusted hazard ratio of hip fracture stratified by sex, age, and comorbidities between the two cohorts.

Variables	Cholangitis						Compared to noncholangitis cohort	
		No			Yes			
	Event	PY	Rate	Event	PY	Rate	Crude HR (95% CI)	Adjusted HR^†^(95% CI)
Overall	366	62466	5.86	101	13332	7.58	1.29 (1.03-1.61)	1.29 (1.03-1.61)
Sex								
Women	218	30211	7.22	61	6619	9.22	1.27 (0.96-1.69)	1.27 (0.96-1.70)
Men	148	32255	4.59	40	6713	5.96	1.29 (0.91-1.83)	1.27 (0.89-1.82)
Age, year								
<65	19	32802	0.58	15	7298	2.06	3.53 (1.80-6.96)	2.65 (1.30-5.39)
≥65	347	29664	11.7	86	6034	14.3	1.22 (0.96-1.54)	1.15 (0.90-1.45)
Comorbidity								
No	29	21929	1.32	9	3348	2.69	2.04 (0.97-4.31)	3.01 (1.42-6.41)
Yes	337	40536	8.31	92	9984	9.21	1.11 (0.88-1.39)	1.26 (1.00-1.59)

PY, person-year; rate, incidence density (per 1,000 person-years); HR, hazard ratio; adjusted HR^†^: multiple analysis including age, sex, each comorbidity, and oral steroid used; CI: confidence interval
